# The Self-Administered Use of Complementary and Alternative Medicine (CAM) Supplements and Antioxidants in Cancer Therapy and the Critical Role of Nrf-2—A Systematic Review

**DOI:** 10.3390/antiox11112149

**Published:** 2022-10-30

**Authors:** Paula Krejbich, Marc Birringer

**Affiliations:** 1 Department of Nutritional, Food and Consumer Sciences, Fulda University of Applied Sciences, Leipziger Straße 123, 36037 Fulda, Germany; 2 Wissenschaftliches Zentrum für Ernährung, Lebensmittel und Nachhaltige Versorgungssysteme (ELVe), Fulda University of Applied Sciences, Leipziger Straße 123, 36037 Fulda, Germany; 3 Public Health Zentrum Fulda, Fulda University of Applied Sciences, Leipziger Straße 123, 36037 Fulda, Germany

**Keywords:** cancer, chemotherapy, radiotherapy, CAM, dietary supplements, vitamins, antioxidants, Nrf2, ROS, drug resistance

## Abstract

Complementary and alternative medicine (CAM) supplements are widely used by cancer patients. Dietary supplements, vitamins and minerals, herbal remedies, and antioxidants are especially popular. In a systematic literature review, 37 studies, each including more than 1000 participants, on CAM, dietary supplement, and vitamin use among cancer patients were identified. Accordingly, cancer patients use antioxidants such as vitamin C (from 2.6% (United Kingdom) to 41.6% (United States)) and vitamin E (from 2.9% (China) to 48% (United States)). Dietary supplements and vitamins are taken for different reasons, but often during conventional cancer treatment involving chemotherapy or radiotherapy and in a self-decided manner without seeking medical advice from healthcare professionals. Drug–drug interactions with dietary supplements or vitamins involving multiple signaling pathways are well described. Since most of the anticancer drugs generate reactive oxygen species (ROS), an adaptive stress response of healthy and malignant cells, mainly driven by the Nrf-2-Keap I network, can be observed. On the one hand, healthy cells should be protected from ROS-overproducing chemotherapy and radiotherapy; on the other hand, ROS production in cancer cells is a “desirable side effect” during anticancer drug treatment. We here describe the paradoxical use of antioxidants and supplements during cancer therapy, possible interactions with anticancer drugs, and the involvement of the Nrf-2 transcription factor.

## 1. Introduction

The term cancer describes a variety of non-communicable diseases defined by the rapid growth of abnormal cells beyond their usual boundaries in different parts of the body [1]. With over 19 million cases and almost 10 million deaths in 2020, cancer is one of the main causes of increased disease burden and is one of the leading causes of death worldwide [1,2,3,4]. Due to early detection abilities and effective treatments, many forms of cancer can be cured with a high probability today [1]. Thus, mortality and survival rates, which depend on several factors, such as country, sex, cancer type and stage, age group, and socio-economic aspects, have improved considerably in recent decades [2,5,6,7]. For instance, the 5-year net survival in case of colorectal cancer increased from 44.2% in 1995 to 60.0% in 2014 in the United Kingdom [8]. Current treatment options for cancer diseases involve surgery, radiotherapy and chemotherapy, hormonal therapies, and biological therapies (such as immunotherapy) [1,4]. Chemotherapy and radiotherapy may be applied before surgery to shrink the tumor or after surgery to suppress further tumor growth and cancer cell metastasis. A regimen may consist of a mono-therapeutic approach or a combination chemotherapy with different drugs that act in a synergistic or additive manner [9].

However, conventional cancer therapies can face obstacles due to drug resistance of abnormal cells [10] and show severe side effects, including pain, fatigue, cognitive issues and neuropathies, anemia, thrombocytopenia and neutropenia, gastrointestinal disorders, hair loss, as well as skin and nail changes [11,12,13]. Side effects are mainly caused by an excessive production of reactive oxygen species (ROS) by the cancer drug or radiotherapy [14]. To overcome barriers and reduce adverse effects of existing therapies, cancer research is focusing on new or complementary therapeutic approaches [15]. In this regard, potentials of antioxidative vitamins (vitamins A, C, and E; carotenoids; and combinations), minerals (selenium and zinc), and phytochemicals (including polyphenols (melatonin, curcumin, epigallocatechin-3-gallate, and resveratrol) and amino acids have been discussed to increase therapeutic efficacy, alleviate side effects of conventional treatments, reverse resistance mechanisms, and reduce systemic toxicity and oxidative stress of chemotherapy and radiotherapy [15,16,17,18,19,20,21,22]. A systematic review by Yasueda et al. [22] investigated the efficacy of antioxidant supplements as adjuvants in cancer therapy. Out of the 49 clinical trials included in this review, only 5 studies (with melatonin) reported an increase in survival rates and 4 studies (also with melatonin) reported an increase in tumor regression rates. Based on the clinical trials assessed, the authors concluded that there is a possibility that antioxidant supplementation might reduce the efficacy of radiotherapy or chemotherapy using anthracyclines, platinum derivatives, or alkylating agents. However, most of the studies reported a reduction in adverse effects using supplements (34 out of 46) during chemo- or radiotherapy [22].

While novel and adjuvant therapies with vitamins, minerals, phytochemicals, or amino acids are still being developed and tested, evidence suggests that relevant substances are already being ingested by cancer patients as part of complementary and alternative medicine (CAM) [23,24,25]. According to the National Cancer Institute of the United States National Institutes of Health, CAM is described as “medical products and practices that are not part of standard medical care” [26], including mind–body therapies, biologically based practices, manipulative and body-based practices, energy healing, and whole medical systems [26]. Due to the complex nature of CAM interventions, evidence on their effectiveness requires alternative holistic approaches [27]. In fact, complementary and alternative medicine (CAM) including supplements is widely used with high levels of satisfaction, while trust in school medicine is fading [28,29,30]. The reasons for that phenomenon are multifaceted, but the use of internet-based search engines (doctor google) as well as the exchange of experiences and recommendations via social networks may foster this trend [28,31,32,33]. At the same time, the global market for over-the-counter (OTC) drugs and supplements grew by 6.8% in 2020 and is expected to continue growing [34]. In addition, the e-commerce market, used for nutritional supplements, nutraceuticals, and botanicals, is increasing [35]. There are numerous studies that show a drug–drug interaction when supplements are simultaneously ingested with prescribed drugs [36,37,38,39,40] or during chemotherapy [41]. Supplements can either interfere with drug metabolism enzymes or drug signaling pathways, and thus increase or inhibit drug activity. However, little is known about the pharmacokinetics and the drug–drug interplay between chemotherapeutics and self-administered adjuvants. 

Through a systematic literature review, this review aims to summarize the self-decided use of CAM supplements, dietary supplements, and antioxidants in cancer patients, and seeks to highlight the potential interference with the Nrf-2 signaling pathway. In addition, the authors discuss the complex interplay between ROS-producing anticancer drugs and the simultaneous use of CAM supplements (CAMSs).

## 2. Systematic Review on the Self-Administered Use of CAM Supplements and Antioxidants by Cancer Patients

The use of CAM by cancer patients has been researched as early as in the 1970s [42]. In an early literature review (26 studies with *n* = 10,690), Ernst and Cassileth [23] reported on the prevalence of CAM therapies among adult cancer patients, ranging from 7 to 64% with an average of 31.4%, often involving specific diets, supplements, and herbal preparations. Another systematic overview of studies published in 1999 (32 studies with *n* = 18,138) found that 64 to 81% of cancer survivors used vitamin or mineral supplements and 26 to 77% used multivitamins [25]. The most recent literature review on the use of CAM therapies by cancer patients identified a total of 152 studies with more than 65,000 participants, reporting a weighted average of 40% for current CAM use (up to 88% in individual studies) and a weighted average of 43% for past CAM use (up to 91%) [24]. In a survey conducted across 14 European countries, 14.8% to 73.1% (average 35.9%) of cancer patients (*n* = 956) reported using 58 CAM therapies which frequently involve herbal remedies as well as vitamins and minerals [43]. Various studies indicate that cancer patients increasingly resort to CAM therapies [24,44,45]. Among different CAM modalities, dietary supplements (such as vitamins and minerals) and herbal remedies (such as plant extracts) are commonly used in Europe and the United States [46], and antioxidants seem to be especially popular, particularly with breast cancer patients [47,48].

To identify recent studies and trends on the use of CAM supplements, including dietary supplements and vitamins, by cancer patients, a systematic literature review was conducted. The methodology of this systematic literature review is described in the following section.

### 2.1. Materials and Methods

Systematic searches for the literature review were performed and completed on 11 January 2022 in PubMed, Web of Science, and ScienceDirect. A combination of the following terms was searched for in the title, abstract, and keywords: (i) “complementary and alternative medicine” OR “CAM” OR “complementary medicine” OR “alternative medicine” OR “complementary and alternative” OR “supplement use” OR “supplement*” OR “vitamin*” OR “antioxidant*” AND (ii) “cancer patient*” OR “cancer survivor*” OR “chemotherapy” OR “radiotherapy” OR “radiation” AND (iii) “survey” OR “questionnaire” OR “trial” OR “cohort”. The results were filtered by publication year (15-year period from 2007 to 2021) as a comprehensive review on vitamin and mineral supplement use by cancer patients, including articles up to December 2006, has been published before [25]. In the database search, a total of 2645 records were identified with 836 records from PubMed, 1476 records from Web of Science, and 333 records from ScienceDirect. After removal of duplicates, 1953 records were screened for inclusion criteria. Included were surveys about the use of complementary and alternative medicine (CAM) and dietary supplements (especially vitamins and antioxidants) in cancer patients (sometimes referred to as cancer survivors) with *n* ≥ 1000 participants, with full text available in English. Systematic reviews and meta-analyses were excluded. Papers were excluded if:

- They focused on the use of CAM providers, modalities requiring a skilled practitioner, or treatments administered by non-medical personnel;
- They investigated dietary patterns, dietary intake (of vitamins and antioxidants), or nutrient status;
- They investigated dietary supplements as a therapy or an intervention in a clinical trial;
- They administered oral nutritional supplements (ONSs) as part of treatment to prevent malnutrition;
- They investigated dietary supplement use in relation to cancer risk or incidence;
- Participants were not cancer patients/survivors (e.g., persons with high cancer risk);
- Information on CAM use was not retrieved from participants (e.g., if it derived from medical records instead of surveys);
- Surveys were conducted with oncologists, nurses, or healthcare professionals (not cancer patients).

In a first screening of the title and abstract according to the inclusion and exclusion criteria, 1535 records which did not fulfill the inclusion criteria were excluded from the review. Through a more detailed screening of the title, abstract, and partly full text (with a special focus on the criterion of ≥1000 participants), a further 361 records which did not fulfill the inclusion criteria were excluded, leaving 57 records for further analysis. After the exclusion of 3 records due to a lack of access to the full text, retrieved full texts of the remaining 54 articles were assessed for eligibility purposes. Then, 17 full-text articles were excluded because (i) participant characteristics did not fulfil the inclusion criteria (e.g., a subgroup of <1000 cancer patients, 2 cohorts each with less than 1000 participants); (ii) CAM/dietary supplements were not included in the CAM definition or not reported in the results (for cancer patients separately); (iii) CAM/dietary supplement use was not surveyed in temporal connection with cancer (>10 years since diagnosis); (iv) the study focused on CAM therapies that required a skilled practitioner, or the classification of CAM use was based on outpatient records/database records about alternative treatments (while the literature review focused on the self-administered use of CAM supplements); (v) dietary intake in general was reported, with no mention of intake from dietary supplements in particular. Finally, 37 articles—15 cross-sectional studies, 13 surveys in cohort studies, and 9 cohort studies—were included in the literature review. The following information was collected from the articles: (i) author name(s) and the year of publication; (ii) the study type, the name of the study or cohort, the number of (cancer) participants, and the country; (iii) information on the study population (the cancer type and basic demographic information); (iv) information on the cancer treatment, i.e., chemotherapy, hormone therapy, radiotherapy, and surgery (if reported); (v) the use of CAM (in relation to the cancer diagnosis); (vi) dietary supplement/vitamin use in general, for specific groups of supplements or single substances; and (vii) selected results highlighted by the authors.

**Figure 1:**
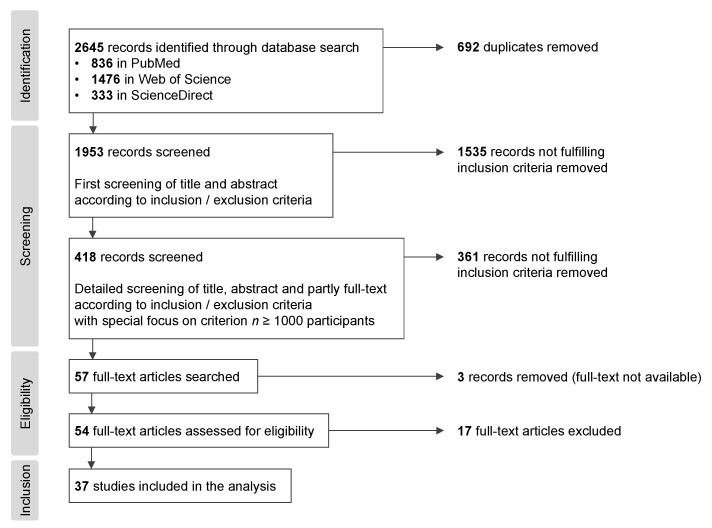
Flow chart of the systematic literature review according to the PRISMA statement [49] (own illustration).

The results of the systematic literature review are presented in Table 1. The steps of the systematic literature search, which was based on the PRISMA statement [49], are illustrated in Figure 1.

### 2.2. Results and Discussion

Table 1 presents the results of a systematic review of literature on CAM use—more specifically dietary supplement and vitamin use—among cancer patients, including studies with a minimum of 1000 participants. Table 1 part A includes cross-sectional studies, surveys in cohort studies, and cohort studies, while part B lists cohort studies in which the use of dietary supplements and vitamins is correlated to cancer prognosis and treatment outcomes. The listed studies investigated patients with various cancer types or with specific diseases, such as breast cancer, who participated in several cohort studies such as the Life After Cancer Epidemiology Study (LACE) [50,51]; the National Health and Nutrition Examination Survey (NHANES) [52]; the Pathways Study [53,54]; the Shanghai Breast Cancer Survival Study (SBCSS) [51,55,56,57]; the Breast Cancer Phase III Trial Comparing Chemotherapy Schedules in High-Risk Early-Stage Breast Cancer (SWOG 0221 (S0221)); the Diet, Exercise, Lifestyle, and Cancer Prognosis Study (DELCaP) [58,59,60]; and the Women’s Healthy Eating and Living Study (WHEL) [61]. Participants frequently reported being, or having been, under cancer treatment (partly specified by population characteristics or predetermined by recruitment), often involving surgery, chemotherapy, hormone therapy, and radiotherapy. Studies investigated CAM use in general or during a specific period, but mainly after diagnosis and during treatment.

**Table 1:** Results of the systematic literature review on CAM, dietary supplement, and vitamin use among cancer patients.

Author	Study Type, Study/Cohort, Participants, Country	Population	Treatment	CAM Use	Dietary Supplement/Vitamin Use		Results
PART A							
Chen et al. [55]	Survey in a cohort study SBCSS 5046 China	Women with breast cancer Average age ^[a]^: 54 years	Chemotherapy: 91.1% of participants Hormone therapy (tamoxifen): 51.9% Radiotherapy: 32.1%	CAM use after diagnosis: 97.2% of participants	Supplement use after diagnosis: 77.2% of participants Melatonin: 0.6% Vitamins: 36.7%		
Conway et al. [62]	Cross-sectional study ASCOT 1049 United Kingdom	Cancer patients recruited from National Health Service sites 38% male, 62% female Mean age: 64 years			Dietary supplement use (24 h dietary recall): 40.0% of participants Multivitamins/minerals: 8.3% Turmeric: 1.9% Vitamin C: 2.6% Vitamin D: 7.7%		
Greenlee et al. [53]	Cohort study Pathways 1000 United States	Women with breast cancer Average age ^[a]^: 60 years	Treatment received 4 to 6 months after diagnosis Chemotherapy: 44.0% of participants Hormone therapy: 40.3% Radiotherapy: 34.3% Surgery: 97.2%	CAM use history: 96.5% of participants CAM use between diagnosis and study enrolment: 86.1%	CAM product use between diagnosis and study enrolment Herbal and botanical supplements ^[b]^: 47.5% of participants Green tea: 40.9% Omega-3 fatty acids: 33.7% Botanicals or other natural products ^[c]^: 63.8% of participants who received chemotherapy		
Greenlee et al. [54]	Cohort study Pathways 2596 United States	Women with breast cancer Median age ^[a]^: 61 years			Vitamin/mineral supplement use after diagnosis: 82.0% of participants Beta-carotene: 1.7% Multivitamins: 60.8% Selenium: 3.1% Vitamin A: 3.1% Vitamin C: 24.7% Vitamin D: 43.1% Vitamin E: 11.6%		Average doses of vitamin/mineral supplements excessed IOM dietary reference intakes by far notable increases in the mean consumption of certain vitamin/mineral supplements after diagnoses among continuous users
Huang et al. [56]	Survey in a cohort study SBCSS 1047 China	Women with breast cancer Mean age ^[a]^: 54 years	Former treatment Chemotherapy: 93.1% of participants Hormone therapy (tamoxifen): 47.5% Radiotherapy: 33.0% Surgery: 99.5%		Regular ^[d]^ supplement use Multivitamins: 10.5% of participants Vitamin A: 1.2% Vitamin C: 6.5% Vitamin D: 0.6% Vitamin E: 2.9%		
John et al. [63]	Cross-sectional study NHIS 2012 2977 ^[e]^ United States	Cancer survivors39% male, 61% female		CAM use during past 12 months CAM (other than vitamins/minerals): 37.9% of participants CAM and/or vitamins/minerals: 78.5%	Vitamins and mineral use during past 12 months: 40.5% of participants		Cancer survivors were more likely to report use of CAM therapies including vitamins/minerals than cancer-free individuals
Kristoffersen et al. [64]	Survey in a cohort study Tromsø study 2015–2016 1636 ^[e]^ Norway	Cancer patients and survivors 47% male, 52% female Mean age: 68 years (patients) and 65 years (survivors)		CAM ^[f]^ use during past 12 months: 29.0% of participants	CAM supplement use during past 12 months Herbal medicines/natural/herbal remedies: 17.4% of participants		No difference in overall CAM use between cancer patients, cancer survivors, and cancer-free individuals
Laengler et al. [65]	Cross-sectional study (retrospective) 1063 Germany	Pediatric cancer patients ^[g]^ recruited from a cancer registry		CAM use after diagnosis: 34.5% of participants Biologically based practices: 18.2%	Dietary supplement use after diagnosis: 12.2% of participants Megavitamins ^[h]^: 3.1%		
Lapidari et al. [66]	Survey in a cohort study CANTO 5237 France	Women with breast cancer Mean age: 56 years	Chemotherapy: 54.0% of participants Hormone therapy: 80.1% Radiotherapy: 90.6% Surgery (breast): 99.9%	Oral CAM ^[i]^ use At or after diagnosis: 23.0% of participants At diagnosis: 11.3% After diagnosis: 11.6% (13.3% of 2829 receiving chemotherapy, 11.8% of 4743 receiving radiotherapy)	Use at or after diagnosis Dietary supplements: 5.4% of participants Herbal supplements: 2.4% Vitamins/minerals: 5.6%		
Lee et al. [67]	Cross-sectional study 1852 South Korea	Cancer survivors recruited from cancer survivor clinics 31% male, 69% female	Chemotherapy: 42.7% of participants Hormone therapy: 27.4% Radiotherapy: 35.6% Surgery: 98.8%		Long-term ^[j]^ dietary supplement use: 15.7% of participants (17.1% of 791 receiving chemotherapy, 19.1% of 660 receiving radiotherapy) Multivitamins: 6.9% of participants Omega-3 fatty acids: 3.7% Vitamin C: 5.0% Vitamin D: 3.3%		
Li et al. [52]	Cross-sectional study (serial) NHANES 1999–2014 4023 ^[e]^ United States	Cancer survivors 41.8% male, 58.2% female			Botanical dietary supplement use during past 30 days: 15.5 to 23.6% of participants, 18.8% of participants from 1999 through 2014 in total		Higher prevalence of botanical dietary supplement use among patients with cancer in each NHANES cycle
Loquai et al. [68]; Loquai et al. [69]	Cross-sectional study 1089 Germany	Patients with melanoma recruited from skin cancer centers 46% male, 46% female Mean age: 59 years	Former or current treatment (specified information): 30.8% BRAF-inhibitor: 2.7% Chemotherapy: 2.6% Interferon: 23.8% Ipilimumab: 0.6% Radiotherapy: 3.7%	Current CAM use: 41.0% of participants Biological-based CAM ^[k]^: 25.9% (28.1% of 335 with former or current treatment)	Current CAM supplement use Chinese herbs and teas: 6.4% of participants Dietary supplements: 14.9% Selenium: 6.8% Vitamins: 10.4%		7.3% of participants (23.9% of 335 with former or current treatment) were at risk of interactions between biological-based CAM and cancer treatment
Luc et al. [70]	Cross-sectional study 5418 ^[e]^ United States	Cancer patients registered in the DBBR 40% male, 60% female			Supplement use at enrolment Multivitamins: 50.6% of participants Supplement use during past ten years Beta-carotene: 4.1% of participants Lutein: 2.8% Lycopene: 2.0% Melatonin: 3.0% Selenium: 5.6% Vitamin A: 7.9% Vitamin C: 33.0% Vitamin D: 27.4% Vitamin E: 24.8%		Higher prevalence of supplementation among cancer-free controls
Mao et al. [71]	Cross-sectional study NHIS 2002 1904 ^[e]^ United States	Cancer survivors 38% male, 62% female		CAM use during past 12 months: 39.8% of participants Biological-based CAM ^[l]^: 21%	CAM supplement use during past 12 months Megavitamin: 4.4% of participants Natural products/herbs: 19.4%		Higher prevalence of CAM among cancer survivors (similar to other participants with chronic illnesses)
Mao et al. [72]	Cross-sectional study NHIS 2007 1471 ^[e]^ United States	Cancer survivors 42% male, 59% female		CAM use during past 12 months: 43.3% of participants Biological-based CAM: 26.0%	CAM supplement use during past 12 months Herbs: 23.2% of participants		Higher prevalence of CAM among cancer survivors
Micke et al. [73]	Cross-sectional study 1013 Germany	Cancer patients receiving radiotherapy recruited from radiotherapy centers 53% male, 47% female Median age: 60 years	Radiotherapy: 100% of participants ^[m]^	CAM use during last 4 weeks before treatment: 59.0% of participants	Supplement use before treatment ^[n]^ Selenium: 10% of participants Vitamins: 18%		
Miller et al. [74]	Cross-sectional study CHIS 2001 1844 ^[e]^ United States	Cancer patients 33% male, 67% female			Dietary supplement use during past 12 months Herb or botanical: 41.0%/48.9% of 268 cancer only participants/1576 cancer patients with chronic illness Multivitamin: 44.1%/53.0% of 268/1576 Single-vitamin: 54.9%/66.3% of 268/1576		Higher prevalence of supplement use in adults with cancer or other chronic conditions
Miller et al. [75]	Survey in a cohort study Penn State Survivor Study 1233 United States	Cancer survivors33% male, 67% female Mean age: 55 years			Regular ^[o]^ dietary supplement use during past month: 73.0% of participants Antioxidants ^[p]^: 40% Calcium/vitamin D: 40% Herbal preparations: 21% Multivitamin-multimineral: 62%		
Pedersen et al. [76] ^[q]^	Survey in a cohort study Nationwide cohort study of Danish women treated for early-stage breast cancer 3343 Denmark	Women with breast cancer treated with surgery Median age: 56 years	Chemotherapy (CEF or CMF) (current): 43.2% of participants Hormone therapy (TAM or TAM + FEM): 62.2% (37.6% current) Radiotherapy: 79.1% (43.8% former) Surgery: 99.8% ^[m]^	CAM use after diagnosis: 40.1% of 3254 participants ^[r]^ (49.4% of participants with current chemotherapy; 32.2% of participants with former radiotherapy)	CAM product use after diagnosis Dietary or vitamin supplements: 27.5% of 3254 participants Herbal medicine: 9.6% of 3254		
Pedersen et al. [77]	Survey in a cohort study Nationwide cohort study of Danish women treated for early-stage breast cancer 2920 Denmark	Women with breast cancer treated with surgery	Treatment received Chemotherapy: 41.9% of participants Radiotherapy: 78.7% Hormone therapy: 64.4% Surgery: 100% ^[m]^	CAM use ^[s]^ since participating in first survey: 49.8% of participants	CAM supplement use since participating in first survey Dietary/nutrition supplements: 31.0% of participants Herbal medicine: 11.3%		Higher prevalence of CAM use in believers
Pouchieu et al. [78]	Survey in a cohort study NutriNet-Santé Study 1081 France	Cancer survivors 32% male, 68% female Average age: 60 years			Dietary supplement use after diagnosis: 51.4% of participants Current dietary supplement use: 40.9% Beta-carotene: 4.3% Lutein: 2.9% Lycopene: 0.8% Omega-3 fatty acids: 5.2% Polyphenols: 7.5% Retinol: 5.6% Selenium: 10.6% Vitamin C: 16.2% Vitamin D: 23.2% Vitamin E: 14.7% Zeaxanthin: 1.2% Other herbal supplements: 3.1%		7 to 8% of 1081 participants (18% of 442 participants with current use of dietary supplements) reported practices with potential adverse effects
Rosen et al. [79]	Cross-sectional study 1327 United States	Patients with thyroid cancer 11% male, 89% female of 1266 participants ^[t]^ Mean age: 47 years		CAM use (except prayer/multivitamins): 74.3% of 1266 participants	CAM supplement use Herbal supplements: 18.5% of 1327 participants Herbal tea: 25.0% Multivitamin/megamultivitamin: 48.4%		
Tank et al. [80]	Cross-sectional study 1217 Germany	Cancer patients recruited from ambulatory cancer care centers 49% male, 51% female Average age: 68 years	Treatment received Oncological medication: 71.9% of participants Radiotherapy (only): 2.4% Surgery (only): 4.6%		Dietary supplement use at study entrance: 47.2% of participants Herbal and botanical supplements: 12.6% of participants Multivitamins: 12.0% Omega-3 fatty acids: 5.7% Selenium: 4.1% Vitamin C: 9.4% Vitamin D: 10.9% Vitamin E: 3.4%		
Velentzis et al. [81]	Survey in a cohort study DietCompLyf study 1560 United Kingdom	Breast cancer patients 100% female	Treatment received Chemotherapy: 46.2 to 51.9% ^[u]^ of participants Hormone therapy: 85.3% Radiotherapy: 85.6 to 91.3% ^[u]^ Surgery: 94.3 to 100% ^[u]^		Dietary supplement use after diagnosis: 62.8% of participants Multivitamins and minerals: 33.7% Estrogen botanical supplements: 8.4% Vitamin C: 14.6%		Significant increase in the use of supplements, multivitamins and minerals, vitamin C, and estrogen botanical supplements after diagnosis
Walshe et al. [82]	Survey in a cohort study Cancer Survival Study 1323 Australia	Cancer survivors 58% male, 41% female Median age: 63 years	Treatment received Chemotherapy: 32.8% of participants Hormone therapy: 16.6% Radiotherapy: 28.8% Surgery: 71.5%	Use of biologically based CAM ^[v]^ in relation to cancer diagnosis or treatment: 26.4% of participants	Use in relation to cancer diagnosis or treatment Herbal treatments: 8.0% of participants Nutritional supplements or vitamins: 23.1%		Higher prevalence of biologically based CAM use among survivors who received chemotherapy, radiotherapy, or other treatments
Yalcin et al. [83]	Cross-sectional study 1499 Turkey	Cancer patients recruited from an outpatient clinic 28% male, 72% female	Treatment received Chemotherapy: 90% of participants Radiotherapy: 53% Surgery: 70%	CAM use: 95.7% of participants	CAM product use: 4.0% of participants Herbal preparations: 2.8% Vitamins: 0.7%		
Zirpoli et al. [59]	Survey in a cohort study S0221 1249 United States	Patients with breast cancer under treatment 100% female Mean age ^[w]^: 51 years	Treatment: 100% of participants ^[m]^		Supplement use during treatment Multivitamins: 43.2% of 1238 participants Omega-3 fatty acids ^[x]^: 12.6% of 1234 Vitamin C: 11.9% of 1238 Vitamin D: 25.4% of 1239 Vitamin E: 6.4% of 1238		
Zirpoli et al. [60]	Survey in a cohort study S0221/DELCaP 1225 (1068 completing the second questionnaire) United States	Breast cancer patients under treatment	Treatment received Chemotherapy: 100% of participants ^[m]^		Dietary supplement use during chemotherapy Multivitamin: 44.4% of 1062 participants Omega-3 sources: 13.0% of 1062 Vitamin C: 12.5% of 1060 Vitamin D: 24.8% of 1061 Vitamin E: 6.9% of 1060		
Zuniga et al. [84]	Survey in a cohort study (serial) CaPSURE 7989 United States	Patients with prostate cancer Average age ^[a]^: 66 years		CAM use after diagnosis: 56% of participants Oral CAM ^[y]^ use: 50%	CAM supplement use after diagnosis Vitamins/minerals: 50% of participants Antioxidants: 32% Herbs: 24% Green tea: 11% Multivitamins: 40% Omega-3 fatty acids ^[z]^: 24% Selenium: 8% Vitamin A: 6% Vitamin C: 17% Vitamin D: 21% Vitamin E: 15%		Increase in overall CAM use, use of multivitamins (minor), and use of omega-3 fatty acids Decrease in use of vitamin E, selenium, and lycopene
PART B							
Ambrosone et al. [58]	Cohort study DELCaP (S0221) 1134 United States	Patients with breast cancer receiving chemotherapy	Chemotherapy: 100% of participants ^[m]^ Cyclophosphamide, doxorubicin, paclitaxel	Supplement use during treatment Antioxidants ^[aa]^: 17.7% of 1132 participants Carotenoid: 1.0% of 1134 Melatonin: 2.1% of 1132 Multivitamins: 43.8% of 1134 Omega-3 fatty acids: 12.6% of 1134 Vitamin A: 2.3% of 1134 Vitamin C: 12.2% of 1134 Vitamin D: 24.6% of 1134 Vitamin E: 6.7% of 1134		Antioxidants ↑ risk of recurrence (*p* = 0.06) Antioxidants ↑ mortality (*p* = 0.14) Vitamin B12 ↑ risk of recurrence * (*p* < 0.01) Vitamin B12 ↑ mortality * (*p* < 0.01) Iron (during chemotherapy) ↑ risk of recurrence * (*p* < 0.01)	
Greenlee et al. [50]	Cohort study LACE 2264 United States	Women with breast cancer with completed treatment Average age: 58 years	Completed treatment: 100% of 2254 participants ^[m]^ Chemotherapy: 57.2% Hormone therapy: 80.5% Radiotherapy: 63.0%	Antioxidant-containing supplement ^[ab]^ use after diagnosis: 80.8% of participants Beta-carotene: 6% Combination carotenoids: 7% Lycopene: 1% Multivitamins: 70% Selenium: 7% Vitamin C: 40% Vitamin E: 48%		Vitamin C ^[ac]^ ↓ risk of recurrence * (*p* = 0.03) Vitamin E ^[ac]^ ↓ risk of recurrence * (*p* = 0.02) Vitamin E ^[ac]^ ↓ all-cause mortality * (*p* = 0.05) Carotenoids ^[ac]^ ↑ breast cancer mortality * (*p* = 0.01) Carotenoids ^[ac]^ ↑ all-cause mortality * (*p* = 0.01)	
Inoue-Choi et al. [85]	Cohort study IWHS 2118 United States	Cancer survivors 100% female Average age: 79 years	First cancer treatment Chemotherapy: 16.8% of participants Hormone therapy: 22.5% Immunotherapy: 2.3% Radiotherapy: 22.2% Surgery: 93.3% Current treatment: 11.0%	Dietary supplement use during the past 12 months: 84.6% of participants Beta-carotene: 2.3% Multivitamins: 63.8% Selenium: 4.2% Vitamin A: 5.2% Vitamin C: 27.0% Vitamin D: 12.0% Vitamin E: 31.0%		Supplements, multivitamins - mortality Multivitamins ↓ mortality * (high diet quality) (*p* = 0.02) Multivitamins + other supplements ↑ mortality * (low diet quality) (*p* = 0.02) Folic acid ↑ mortality * (low diet quality) (*p* = 0.006)	
Nechuta et al. [57]	Cohort study SBCSS 4877 China	Women with breast cancer	Treatment received within 6 months after diagnosis Chemotherapy: 92.2% of participants Hormone therapy (tamoxifen): 51.7% Radiotherapy: 32.7%	Vitamin supplement use after diagnosis: 36.4% of participants (29.8% of 4497 during chemotherapy; 26.2% of 1597 during radiotherapy) Antioxidants ^[ad]^: 28.3% (22.2% of 4497 during chemotherapy; 20.9% of 1597 during radiotherapy) Multivitamins: 11.0% Vitamin A: 1.7% Vitamin C: 15.3% Vitamin D: 0.4% Vitamin E: 6.1%		Vitamins ^[ae]^ ↓ risk of recurrence * (*p* = 0.06) Vitamins ^[af]^ ↓ mortality * (*p* = 0.05) Antioxidants ^[ae]^ ↓ risk of recurrence * (*p* = 0.02) (participants with no radiotherapy) Antioxidants ^[af]^ ↓ mortality * (*p* = 0.001) (participants with no radiotherapy) Vitamin E ^[ae]^ ↓ risk of recurrence * (*p* = 0.04) Vitamin E ^[af]^ ↓ mortality * (*p* = 0.05) Vitamin C ^[af]^ ↓ risk of recurrence * (*p* = 0.01) Vitamin C ^[af]^ ↓ mortality* (*p* = 0.009)	
Poole et al. [51]	Cohort study ABCPP: SBCSS, LACE, WHEL, NHS 12,019 United States, China	Breast cancer survivors 100% female Mean age ^[a]^: 57 years		Regular ^[ag]^ supplement use after treatment: 60.6% of participants Multivitamins: 16.6% (65% of 1999 multivitamin users received chemotherapy; 56% received radiotherapy) Any other single supplement ^[ai]^: 43.9% (60% of 5279 single-supplement users received chemotherapy; 56% received radiotherapy)		Vitamins ^[ae]^–risk of recurrence Vitamins ^[ae]^–mortality Antioxidants ^[ah]^ ↓ all-cause mortality *	
Saquib et al. [61]	Cohort study WHEL 2562 United States	Breast cancer survivors 100% female	Prior systemic treatment: 94.3% of 3086 WHEL participants	Dietary supplement use during past 24 h: 85% of participants Antioxidant: 9.8% of 2909 WHEL participants receiving systemic treatment Herbals: 26.0% Herbals (phytoestrogens): 6.9% Multivitamin/mineral: 52.9% Vitamin A: 1.7% Vitamin C: 41.6% Vitamin D: 1.8% Vitamin E: 46.0%		CAM/supplements–risk of recurrence (participants who received systemic treatment)	
Skeie et al. [86]	Cohort study Norwegian Women and Cancer cohort study 2997 Norway	Cancer patients with solid tumors 100% female Mean age ^[a]^: 58 years		Dietary supplement use before diagnosis: 47.1% of participants Occasional use: 10.6% Daily use: 36.5%		Dietary supplements ^[aj]^ ↓ mortality * (lung cancer patients)	

Abbreviations: ABCPP: After Breast Cancer Pooling Project; ASCOT: Advancing Survival after Cancer Outcomes Trial; CAM: complementary and alternative medicine; CANTO: cancer toxicities; CaPSURE: Cancer of the Prostate Strategic Urologic Research Endeavor; CHIS: California Health Interview Survey; DBBR: Data Bank and BioRepository; DELCaP: Diet, Exercise, Lifestyle, and Cancer Prognosis Study; IOM: Institute of Medicine; IWHS: Iowa Women’s Health Study; LACE: Life After Cancer Epidemiology Study; NHANES: National Health and Nutrition Examination Survey; NHIS: National Health Interview Survey; NHS: Nurses’ Health Study; NHS II: Nurses’ Health Study II; SBCSS: Shanghai Breast Cancer Survival Study; S0221: SWOG 0221/Breast Cancer Phase III Trial Comparing Chemotherapy Schedules in High-Risk Early-Stage Breast Cancer; WHEL: Women’s Healthy Eating and Living Study. ^[a]^ at diagnosis; ^[b]^ botanical supplements: green tea, Echinacea, flax seed, cranberry, chamomile, garlic, ginseng, and ginger; ^[c]^ other natural products: glucosamine, fish oils, omega-3 fatty acids, laxatives, chondroitin, fiber supplement, and acidophilus; ^[d]^ at least once a week for more than one month; ^[e]^ cancer participants in a larger study population; ^[f]^ traditional and complementary medicine (T&CM); ^[g]^ survey answered by parents; ^[h]^ megadose vitamins; ^[i]^ oral CAM: homeopathy, or vitamins/minerals, or herbal supplements, or other dietary supplements; ^[j]^ at least 6 months; ^[k]^ biological-based CAM: vitamins, trace elements, other supplements, and phytotherapeuticals as Chinese herbs; ^[l]^ biological-based CAM: “CAM products such as herbs and megavitamins that were believed to have biological efficacy”; ^[m]^ population characteristics; ^[n]^ during last 4 weeks before treatment; ^[o]^ at least once a week; ^[p]^ antioxidants: β-carotene, vitamins (A, C, or E), and antioxidant vitamin combination supplements; ^[q]^ treatment data published by Christensen et al. [87]; ^[r]^ participants with complete CAM data; ^[s]^ CAM use: alternative treatments; ^[t]^ participants included in logistic regression; ^[u]^ category “other combinations”; ^[v]^ biologically based CAM: herbs, dietary supplements, vitamins, minerals, botanicals, probiotics, whole diets, and functional foods; ^[w]^ at baseline; ^[x]^ omega-3 fatty acids: fish oil, EPA, omega-3, flaxseed, or cod liver oil; ^[y]^ oral CAM: “ingesting CAM” (not including multivitamins); ^[z]^ omega-3 fatty acids from flaxseed oil, fish oil, or other supplements; ^[aa]^ antioxidants: vitamins A, C, and E; carotenoids; coenzyme Q10; ^[ab]^ antioxidant-containing supplement: multivitamins, combination carotenoids, vitamin C, vitamin E, beta-carotene, lycopene, selenium, and zinc; ^[ac]^ frequent use (6–7 days per week); ^[ad]^ antioxidants: vitamin E, vitamin C, and multivitamins; ^[ae]^ after diagnosis; ^[af]^ after diagnosis for >3 months; ^[ag]^ at least 1 year; ^[ah]^ antioxidants: multivitamins, vitamin C, or vitamin E; ^[ai]^ single supplement: vitamins A, B, C, D, and E; ^[aj]^ daily/occasional. ↑ increase, ↓ decrease. * significant effect.

#### 2.2.1. CAM Supplement Use by Cancer Patients

The use of CAM therapies after diagnosis was reported to be as high as 97.2% [55] and 86.1% of participants reporting CAM use [53], and up to 49.4% of participants during chemotherapy [76]. After diagnosis, supplements were used by up to 77.2% of participants [55] and vitamin or mineral supplements were used by up to 82.0% of participants [54]. The intake of multivitamins after diagnosis was common, with up to 70% [50] or 60.8% of participants taking usage [54]. Single supplements were also frequently used by up to 43.9% of participants [51], and use of botanicals after diagnosis was reported by up to 47.5% of participants [53]. Supplements containing antioxidants were equally popular among cancer patients, with use after diagnosis confirmed by as many as 80.8% of participants [50]. The use of dietary supplements and vitamins was often reported during conventional treatment including chemo- and radiotherapy. According to Zirpoli et al. [59], up to 43.2% of participants reported the intake of multivitamins during cancer treatment. Concurrently with chemotherapy, vitamin supplements were used by 29.8% and antioxidants were used by 22.2% of cancer patients [57], while multivitamin use during chemotherapy was prevalent in 43.8% of participants enrolled in another study [58]. The literature suggests that cancer patients rethink their lifestyle in relation to dietary supplement and vitamin use after their diagnosis. For instance, Greenlee et al. [54] report that 60.2% of participants started using vitamins or mineral supplements, while 46.3% discontinued using supplements. Similarly, in a study by Tank et al. [80], where 41.5% of supplement users initiated their routine after their disease was diagnosed, it was common for patients to start using dietary supplements after diagnosis. However, other articles reported lower rates, such as 15.3% of participants [81] or 14% of participants beginning (dietary) supplement use after their diagnosis [78].

The studies in Table 1 reported the following use of individual substances (differences in timing of intake possible): beta-carotene was used by 1.7% (United States) to 6% (United States) of participants (*n* = 5 studies); omega-3 fatty acids was used by 3.7% (South Korea) to 33.7% (United States) of participants (*n* = 8); selenium was used by 3.1% (United States) to 10.6% (France) of participants (*n* = 9); vitamin A was used by 1.2% (China) to 7.9% (United States) of participants (*n* = 8); vitamin C was used by 2.6% (United Kingdom) to 41.6% (United States) of participants (*n* = 16); vitamin D was used by 0.4% (China) to 43.1% (United States) of participants (*n* = 15); and vitamin E was used by 2.9% (China) to 48% of participants (United States) (*n* = 13).

As observed in previous studies, the use of CAM is most prevalent among younger and female individuals with higher educational levels, and especially popular among breast cancer patients [46]. Numerous studies have suggested that women with breast cancer are particularly prone to CAM use [47,48,76,88]. The decision made by cancer patients to use CAM therapies is described as “a nonlinear, complex, dynamic process” [89], influenced by a variety of factors which occur in different periods, including an early phase after diagnosis, an intermediate phase, and an end phase after conventional treatment [89]. According to different studies, cancer patients use CAM to sustain physical well-being by reducing symptoms of their disease and alleviating side effects of conventional treatments to actively participate in and contribute to the cure of their cancer by supporting their body and immune system, to prevent later recurrence of the disease, or even to control the growth of abnormal cells and cure their cancer [46,47,90]. Considering a possible dissatisfaction with conventional therapies, the decision for alternative treatments—which are misperceived as safe therapies with few side effects—also reflects a desire for less toxic treatments [28,33,91]. 

Cancer patients often report high levels of satisfaction with the use of CAM therapies [29,30], which are perceived as beneficial and effective approaches used to treat their cancer disease [76]. For instance, in a survey, Alsanad et al. [92] found that around 75% of dietary supplement users reported benefits, mainly attributed to supplement intake. In another study, most participants reporting CAM use (such as dietary interventions) considered their CAM treatment to be helpful and were willing to recommend such therapies to others [47]. Indeed, recommendations from other persons may influence the decision of cancer patients to use CAM. Accordingly, motivations surrounding CAM use were mainly based on recommendations of family or friends in a nationwide survey in Japan by Hyod et al. [93]. Besides recommendations from family members and friends, other studies identified the internet, social networks (such as Facebook), and media as important information sources [28,31,32,33], as well as non-scientific literature, lay press, and newspapers, especially after receiving insufficient medical advice from their physician [91]. Studies also suggest a high level of trust in the information, regardless of their source [33]. This finding is concerning given that CAM therapies, in general, are more affordable than conventional treatments, and that dietary supplements as OTC drugs are available without prescription and therefore are easily accessible, especially in e-commerce, representing an important sales channel for dietary supplements [28,46,88,94].

Consequently, various studies indicate that cancer patients frequently use CAM and dietary supplements without seeking medical advice and even without informing healthcare professionals or their treating oncologists. Patients may consider information on CAM use as irrelevant and may fear the rejection of dietary supplements or the non-consideration of personal preferences by their physicians [91,95]. In addition, overall poor communication is assumed to be a contributing factor, which is discussed in detail by Frenkel and Cohen [96]. This is a critical issue as the literature suggests that CAM therapies, especially dietary supplements and vitamins, are used concurrently with conventional treatments such as chemotherapy and radiotherapy (Table 1). However, the use of dietary supplements while receiving chemotherapy or radiotherapy is generally not recommended for cancer patients [97].

#### 2.2.2. Possible Adverse Effects of CAM Supplement Use by Cancer Patients

While evidence on benefits of CAM supplements for cancer patients remains inconclusive [45,48,98], possible negative consequences and adverse effects have been proposed by several studies. On the one hand, a survey conducted in a study cohort found dietary supplement use in breast cancer patients to be associated with the non-initiation of clinically indicated chemotherapy [98]. On the other hand, possible interactions of dietary supplements and antioxidants with reduced effectiveness of conventional treatment have been assumed in the literature [88,92,99]. For instance, Loquai et al. [69] investigated the use of CAM among melanoma patients and concluded that 7.3% of all participants (*n* = 1089) and 23.9% of participants with specified information on former or current treatment (*n* = 335) were at risk of interactions between cancer treatment and biological-based CAM (Table 1, part A). A similar study on cancer survivors enrolled in the NutriNet-Santé study (*n* = 1081) found that 18% of participants currently using dietary supplements (*n* = 442) are engaged in supplementation practices with potential harmful effects [78] (Table 1, part A). These findings are supported by further studies that were not included in the systematic literature review due to lower participant numbers. Firkins et al. [100] surveyed CAM and dietary supplement use among cancer patients under treatment and, through a literature-based evaluation of potential interactions, found that 15.9% of participants (*n* = 711) were at risk of interaction between anticancer medication and biological-based CAM (such as vitamins A, C, and E). In a smaller group of cancer patients (*n* = 115), another study identified possible interactions with conventional treatments in 51.2% of participants using CAM supplements, such as vitamins and minerals (*n* = 43), evaluated as likely in 37.2% of participants [46,101]. The authors comprehensively describe potential adverse effects of individual CAM supplements in cancer therapy but highlight the theoretical nature of their findings and emphasize the need for further research including clinical studies [46,101], which is also supported by other studies [88].

The effect of dietary supplements on primary outcomes, such as cancer recurrence and mortality, mainly in breast cancer patients, has been investigated in several association studies which, however, also provide inconclusive results (Table 1, part B). Thus, vitamin supplement use after diagnosis was associated with a lower risk of recurrence (*p* = 0.06) and lower mortality (*p* = 0.05) [57] or showed no association with recurrence and mortality [51]. Though supplement and multivitamin use after diagnosis was not associated with mortality in general, Inoue-Choi et al. [85] found multivitamins to be associated with lower mortality in participants with high diet quality (*p* = 0.02), while the intake of multivitamins together with other supplements was associated with higher mortality in participants with low diet quality (*p* = 0.02). With regard to single vitamins, Greenlee et al. [50] investigated the use of antioxidant-containing supplements after diagnosis and concluded that the intake of vitamin C (*p* = 0.03) and vitamin E (*p* = 0.02) was associated with a lower risk of recurrence, and vitamin E was also associated with lower all-cause mortality (*p* = 0.05), while cancer-specific (*p* = 0.01) and all-cause mortality (*p* = 0.01) was higher with the combined intake of carotenoids. These findings are supported by Nechuta et al. [57], who found vitamin C and vitamin E to be associated with a lower risk of recurrence (*p* = 0.01 and *p* = 0.04, respectively) and lower mortality (*p* = 0.009 and *p* = 0.05, respectively). Furthermore, earlier association studies showed a positive effect of antioxidants on all-cause mortality [51], as well as on risk of recurrence (*p* = 0.02) and mortality (*p* = 0.001) in patients not receiving radiotherapy [57]. However, more recent cohort studies with breast cancer patients indicate the adverse effects of antioxidant use, especially during chemotherapy or radiotherapy. In a study with 1134 breast cancer patients, Ambrosone et al. [58] found the use of antioxidants to be associated with a higher risk of recurrence (*p* = 0.06) and higher mortality (*p* = 0.14). A similar finding was reported by Jung et al. [48] in a cohort study with 2223 breast cancer patients (not listed in Table 1), who found that taking antioxidants during chemotherapy or radiotherapy reduced recurrence-free survival (*p* = 0.001 and *p* = 0.02, respectively), and that taking antioxidants during radiotherapy even increased all-cause mortality (*p* = 0.04).

Considering these inconclusive findings discussed in the literature [22,48], the intake of supplements by cancer patients, especially during their conventional treatment, seems at least questionable. According to different authors, dietary supplements, and especially antioxidants taken during conventional treatment, may exert various effects by reducing the toxicity of conventional anticancer therapies (and thus their side effects), but in consequence also by reducing the effectiveness of anticancer drugs and radiotherapy [46,48,88,99]. In fact, chemotherapeutic agents and radiotherapy exert their effectiveness by producing ROS, increasing oxidative stress in cancer cells. On the contrary, antioxidants such as vitamins (A, C, and E), minerals, and polyphenols reduce ROS, thus not only protecting normal cells, but also potentially cancer cells from oxidative stress [22,46,88,99]. Based on this mechanism, Andersen et al. [99], who investigated antioxidant use in cancer patients receiving chemotherapy, found that more than one-quarter of participants treated with anthracyclines (doxorubicin) and platinum-based anticancer drugs (carboplatin and cisplatin) were at potential risk of reduced effectiveness due to antioxidants. An even higher proportion of possibly compromised anticancer therapies was found in a similar newer study [88]. While adverse effects caused by antioxidants during cancer treatment have been suggested before, they were mainly based on theoretical knowledge [46,88,92,99], except for the use of antioxidants during radiotherapy in smokers [102]. However, the theoretical nature of this relationship was recently changed by the evidence presented by Ambrosone et al. [58] and Jung et al. [48], which contradicts earlier findings (e.g., those of Poole et al. [51]). These findings provide a reasonable basis for a more detailed investigation of possible mechanisms involved in the interaction between dietary supplements and antioxidants used in CAM therapies and conventional cancer treatment. In the following sections, the possible mechanisms of interaction between anticancer drugs (exerting their effect through ROS) and antioxidants in the context of the Nrf2 pathway will be reviewed in detail.

## 3. The Critical Role of Nrf-2-Keap I in the Interplay between CAM Supplements and Cancer Therapy

### 3.1. The Nrf-2-Keap I System in ROS Homeostasis and Cancer Drug Resistance

Malignant cells are characterized by distinct physiological and morphological differences from normal tissue. To name a few, accelerated cell cycles, genomic alterations, vascularizations in normal tissue, and hypoxic conditions (in solid tumors) are typical indicators of tumor tissue [103]. The hypoxic environment of solid tumors leads to an increased production of ROS, and thus further modifications of DNA [104]. In addition, programmed cell death by apoptosis is missing in cancer cells, leading to an uncontrolled growth of the tissue. Several transcription factors were identified to play a major role in the adaptation of tumor cells to these conditions. Predominantly, the hypoxia-inducible factor (HIF) family and nuclear erythroid-related factor-2 (Nrf-2) coordinate an adaptive stress response, whereas signal transducers and activators of transcription 3 (STAT3) and nuclear factor kappa-B (NF-κB) are mediators of inflammation. All factors are activated by oncogenic signaling pathways, such as oxidative stress, cytokines, hypoxia, ultraviolet (UV) radiation, and growth factors [104,105]. In this chapter, we focus on the ambivalent role of Nrf-2 in cancer and cancer therapy.

#### 3.1.1. Nrf-2-Keap I as ROS Sensor

Nrf-2 is a primary transcription factor that enables a cellular defense against xenobiotics, such as drugs or phytochemicals and endogenous ROS. The protein belongs to the basic leucine zipper DNA-binding proteins and binds to the promoter of the antioxidant responsive element (ARE) of genes which belong to the cellular defense. During normal ROS homeostasis, cytosolic Nrf-2 is associated with Kelch-like ECH-associated protein 1 (Keap I), leading to continuous degradation via the 26 s proteasome [106]. Keap I contains numerous cysteine residues that sense changes in redox homeostasis or are chemically attacked by electron acceptors (Michael acceptors) [107]. The generation of ROS (consisting of hydrogen peroxide (H_2_O_2_), hydroxyl radical (OH^·^), and superoxide anion (O_2_^−^)) by endogenous (i.e., inflammation or infection) or exogene (i.e., chemicals or UV radiation) stressors leads to Nrf-2 activation. As stable “end products” of oxidative stress, 4-hydroxynonenal (HNE) from lipid oxidation and 8-hydroxydeoxyguanosine (8-OHdG) from DNA-oxidation are generated. HNE is a strong inducer of Nrf-2 by covalently binding to redox-sensitive cysteine residues in Keap I. In that case, Nrf-2 is detached from Keap I and translocates to the nucleus, where it induces a cellular response. It binds to ARE as a heterodimer with small musculoaponeurotic fibrosarcoma proteins (sMafs). Up to now, more than 40 genes are known to be regulated by the Nrf-2 transcription factor [108]. They belong to the detoxification system of cells with phase I and phase II drug-metabolizing enzymes and phase III drug transporters. Among these, phase I enzymes such as cytochrome P450 (CYPs) or aldo-keto reductases can activate drugs for further processing. Phase II enzymes conjugate the drug to eliminate the glucuronide, glutathione, or sulfate conjugates. Finally, multidrug resistance-associated proteins (MRPs), P-glycoptrotein (P-gp) (otherwise known as multidrug resistance protein 1 (MRD1)), or the organic anion-transporting polypeptide (OATP2) enable the transport of xenobiotics out of the cytosol. In addition, Nrf-2 regulates antioxidative enzymes such as superoxide dismutase (SOD), thioredoxin reductases (TrxRs), peroxiredoxins (Prxs), or glutathione peroxidase (GPX) to counteract the superoxide and peroxide disruption of redox homeostasis. Finally, it influences heme metabolism, NADPH generation, fatty acid synthesis and oxidation, purine synthesis, and the expression of other transcription factors [108]. 

#### 3.1.2. Nrf-2 Dual Role in Cancer

Several outstanding reviews described Nrf-2 as a hallmark of malignant cells [108,109,110,111]. The role of Nrf-2 in cancer development is a double-edged sword. Nrf-2 maintains redox homeostasis in normal cells and thus acts as tumor-suppressive, while it is constitutively activated in many cancer cells to maintain an enhanced resistance against hypoxic conditions. The transcription factor activates pro-survival genes to enhance proliferation, promotes tumor progression and metastasis, and inhibits pro-apoptotic cell signals. From a clinical perspective, patients with a high expression of Nrf-2 in their tumor tissue have a higher risk of recurrence and a poor survival prognosis, mainly due to the increased chemo- and/or radioresistance of the tumor [112,113]. 

There is sufficient physiological evidence for both tumor-suppressing and oncogenic activity. Several experimental designs have shown the anti-carcinogenic activity of Nrf-2 as the protein may prevent tumorigenesis, as seen in Nrf-2 knockout mice which show an increased sensitivity to exogenous chemicals and carcinogens [114]. Moreover, a nucleotide polymorphism in the Nrf-2 promoter region (rs6721961) could increase the risk of lung cancer in current and former smokers [115].

However, oncogenic events can lead to an Nrf-2 overexpression in cancer cells, thus fostering tumor cell survival [115]. Among those, there are somatic mutations in Nrf-2, Keap I, or its adaptor protein cullin 3-containing E3 ubiquitin ligase (CuI3) that disrupt the interaction of Nrf-2 and Keap I and inhibit proteasomal degradation. In addition, the significance of autophagy-related protein p62 is increased when autophagy is blocked. The protein competes with Nrf-2 for Keap I-binding and leads to the prolonged activation of Nrf-2. Finally, an increased transcription of the Nrf-2 gene can occur by epigenetic changes in the Nrf-2 promoter, as well as mutations of the tumor suppressor PTEN or oncogenic mutations of Cmyc, k-Ras, and B-Raf [116]. Several other oncogenic signaling pathways, such as phosphatidylinositol-3-kinase (PI3K), nuclear factor kappa-B (NF-κB), or the antiapoptotic Bcl-2 protein, also modulate Nrf-2 activity [117].

Interestingly, Nrf-2 transcriptional activity seems to depend on the amount of oxidative stress applied to a cell. Zucker et al. [118] showed a differential response to oxidative stress (H_2_O_2_) that is mediated by Krüppel-like factor 9 (Klf9). A low dose of hydrogen peroxide activates typical genes promoted via ARE, while lethal levels of H_2_O_2_-triggered Nrf-2 mediated the transcription of Klf9, which in turn represses thioredoxin reductase-2 and peroxiredoxin-6 expression [118]. In consequence, the ROS levels are further increased and induce apoptosis in the cell. Similar observations were made with low and high doses of sulforaphane (SFN) in human lens epithelial cells [119]. In conclusion, Kfl9 activation seems to represent a switch towards the apoptosis of highly stressed cells; however, malignant cells also face a high amount of ROS and resist pro-apoptotic signals.

#### 3.1.3. Nrf-2 in Cancer Cell Resistance

In general, chemical drug resistance is associated with limited cellular drug uptake, different expression levels, or mutations of drug target or increased drug efflux. Cancer cell resistance is accompanied with the expression of membrane transporter proteins that facilitate drug efflux [120]. The most investigated transporters are MDR1, MRP1, and breast cancer resistance protein (BCRP), which are representatives of the ATP-binding cassette (ABC) transporter family. Liu et al. [121] summarized the transcriptional regulation of efflux transporters by redox-sensitive transcription factors Nrf-2, Forkhead box O (FOXO), and apurinic–apyrimidinic endonuclease 1 (APE-1). In fact, oxidative stress levels of the tumor or ROS induced by chemo- or radiotherapy facilitate drug efflux. In addition, most of the current anti-cancer therapies induce ROS production (compiled in Liu et al. [121] and Section 4) and increase in Nrf-2 mRNA and protein levels. Thus, malignant cells counteract rising ROS levels to acquire a new redox balance with higher ROS levels through up-regulated antioxidant enzyme systems. This concept was named “redox resetting” by Liu et al. [121].

In consequence, the use of Nrf-2 inhibitors as a pharmaceutical intervention to overcome chemo- and radioresistance was suggested [111]. The recent literature is focused on the ambivalence of Nrf-2 in cancer, especially in acquired cancer cell resistance and in its role as a target for pharmaceutical interventions [105,111,122,123,124]. The following chapter discusses the role of ROS and Nrf-2 during cancer therapy and the possible interplay between the Nrf-2 signaling pathway and CAMS.

### 3.2. Nrf-2 Activation by Cancer Drugs and the Role of CAMS

#### 3.2.1. Main Targets of Cancer Drugs and ROS Production as a Side-Effect

As described in several excellent reviews, anticancer therapeutics induce an overproduction of free radicals and/or ROS, and thus affect both cancer cells and normal tissue [121,125,126,127]. For some drugs, ROS production is discussed to be the main mode of action; however, for others, the chemical modification of tissue DNA is the main target, and the production of ROS is a “desirable side effect”. We here present ROS production as an essential and common mode of action in the therapeutic use of anticancer drugs and radiation therapy. We also address the influence of ROS overproduction on the Nrf-2-Keap I-dependent signaling pathway and the role of Nrf-2 activation in cancer cells that may lead to an acquired resistance against drugs [9,124,128,129] (see also Section 3.2).

As each anticancer therapy carries a burden of side effects in searching for remedies, cancer patients often end up using CAMSs. We will present evidence on the concept that many CAMSs activate the Nrf-2 pathway, eventually leading to an adaptive stress response and the protection of healthy cells against ROS-induced side effects such as chemotherapy-induced peripheral neuropathies (CIPN), ototoxicity, cardiotoxicity, and others. However, as mentioned above, Nrf-2 activation also fosters cancer cell resistance and therefore might limit the success of the therapy. This dilemma often leads to a paradoxical use of antioxidants during chemotherapy, either by the doctor’s prescription (reviewed by Yasueda et al. [22]) or self-decided. In Section 2, we reviewed the self-administered use of antioxidants and dietary supplements in cancer therapy. Since we do not exactly know how each single CAMS affects the cancer tissue and how the Nrf-2 pathway is involved, we like to hypothesize the following questions:

1. How do CAMSs, and especially antioxidants, interact with the Nrf-2 pathway during cancer therapy?
2. Do CAMSs induce Nrf-2 activation followed by an adaptive stress response of healthy cells or do CAMSs even help the tumor cells acquire resistance?
3. What lessons did we learn from clinical studies with antioxidants as adjuvants in cancer therapy?
4. In consequence, how do CAMSs interact with anticancer drugs and radiotherapy and influence their success in cancer therapy?

The most frequently used drugs are cis-platinum derivatives, anticancer antibiotics, taxanes, and alkylating agents, respectively [130]. In addition, radiotherapy is used as monotherapy or in combination with the aforementioned cancer drugs. This chapter aims to highlight the main targets of cancer therapies, the role of ROS in killing cancer cells, and the risk of harm to healthy tissue. Further, the involvement of the Nrf-2 signaling pathway during cancer treatment is discussed and the with dietary supplements on the related therapeutic strategies is highlighted.

#### 3.2.2. Anthracyclines

Antitumor antibiotics of the anthracycline type are based on tetracyclic molecules with an anthraquinone core structure. They have been isolated from *Streptomyces* species and used since the 1960s to treat various cancer types, especially those that occur in children and in young people.

It is well documented that anthracyclines, such as doxorubicin (DOX), inhibit topoisomerase II as well as DNA polymerase [131,132,133]. It has been reported that anthracyclines are able to intercalate with DNA, forming DNA strand breaks, DNA adducts, and DNA cross-linking. These modifications inhibit DNA transcription and replication and trigger mitochondrial dysfunction, which consequently leads to apoptotic cell death in proliferating cells. Mitochondrial-induced cell death is also associated with the generation of ROS and the activation of apoptotic signaling cascades. In addition, doxorubicin (otherwise known as adriamycin) directly generates ROS within the mitochondria as it interacts with the NADH dehydrogenase of complex I in the electron transfer chain and generates superoxide anion radicals (O_2_^−^) [131,134]. Among the anticancer drugs discussed in this chapter, DOX seems to generate the highest amount of ROS in cancer cells [14]. In previous experiments, doxorubicin reduced the protein level and enzymatic activity of copper–zinc superoxide dismutase (CuZnSOD) in the heart of rats [135]. In addition, the interaction of doxorubicin with NADPH reductase and endothelial nitric oxide synthase (eNOS), respectively, generates free radicals. In addition, doxorubicin reduces the activity of selenium-dependent glutathione peroxidase in the heart of selenium-depleted mice [136]. Given the fact that cardiomyocytes are rich in mitochondria, it is not surprising that cardiotoxicity is the main side effect of doxorubicin treatment in cancer therapy.

ROS generation induced by drugs or natural products is often accompanied by the activation of the Nrf-2-Keap I-pathway and an antioxidative stress response. In vitro experiments with doxorubicin confirmed this activation [137]; however, a recent study by the same authors suggests that a chronic administration of doxorubicin in rats suppresses Nrf2 activation. Thus, a strong antioxidant response is missing that would finally scavenge and eliminate ROS [138]. Interestingly, the authors found an increase in Keap I protein, leading to a negative regulation of Nrf-2 expression. Considering these in vivo results, the activation of Nrf-2 before doxorubicin administration would induce a stress response that counteracts doxorubicin-mediated cardiotoxicity.

According to Table 1, vitamins A, C, E, and selenium are the most prominent antioxidants reported as single dietary supplements during chemotherapy. We here focus on vitamin C (used by up to 41.6% of cancer patients) and vitamin E (used by up to 48% of cancer patients). The latest Cochrane Library review on cardioprotective interventions in cancer patients receiving anthracyclines finds no evidence of a beneficial effect with a combined vitamin C, vitamin E, and N-acetyl-cysteine (NAC) intervention [139]. Vitamin E as a chemoprotective adjuvant has shown promising results in animal studies; however, dosage and treatment schedule are crucial for its preventive effect. In contrast to these findings, several clinical trials with vitamin E adjuvant therapy failed to protect against DOX-induced cardiotoxicity [140]. In light of a current review by Cavalcanti et al. [141] and a systematic review published by Yasueda et al. [22], we question the effectiveness of the self-decided gavage of antioxidant vitamins in the prevention of DOX-induced side effects.

Ascorbic acid (vitamin C) is one of the strongest water-soluble antioxidants and its use in adjuvant therapy with anticancer drugs is well documented [17,142,143]. Besides numerous studies with cancer cell lines, such as vitamin E, ascorbic acid shows only weak cardio and hepatoprotective effects in rats when treated with DOX (compiled by Granados-Principal et al. [140]). Only few clinical trials were conducted with vitamin C as adjuvant in DOX therapy. Suhail et al. [144] administered vitamin C and E to patients with breast carcinoma and found a significant increase in antioxidant enzymatic activity; however, no effects were observed on survival rates or tumor size changes. In general, vitamin C administration is well tolerated with only few side effects; however, clinical evidence for its use as adjuvant in DOX-therapy is limited.

With regard to the effect of vitamin E and C on Nrf-2 activation, when administered to Caco-2 cells, only delta-tocopherol led to a more or less weak induction of Nrf-2 [145]. The most prominent isoform alpha-tocopherol does not significantly induce this pathway. The co-administration of alpha-tocopherol with As_2_O_3_ in H9c2 cardiomyocytes also induced Nrf-2 mRNA, even more pronounced in combination with vitamin C [146]. Vitamin C shows a biphasic behavior where low concentrations scavenge radicals and high concentrations produce cytotoxic ROS [147]. In parallel, Nrf-2 activation and nuclear translocation has been observed [126,148]. Interestingly, ongoing clinical trials use high-dose intravenous (IV) vitamin C dosing as monotherapy in several cancers, yet high-quality evidence is missing due to a limited numbers of patients [142].

Numerous natural products and antioxidants have been used as adjuvant therapies prior to, or concurrently with, doxorubicin administration [132,140]. In a recent review, Yarmohammadi et al. [134] reviewed the protective effects of natural products against DOX-induced cardiac damage in mice, rats, and cardiomyocytes (H9c2 cells). Several flavonoids showed protective effects against DOX-induced toxicity in animal studies and cancer cell lines [149]. Lin and colleagues reported a reduced cardiotoxicity of DOX in rats when co-administered with Shaoxing rice wine polyphenols [150]. The polyphenols attenuated DOX-induced ROS production via the activation of Nrf-2 antioxidative defense and reduced the levels of pro-fibrotic proteins such as TGF-β1, collagen I, and α-SMA. An extract of *Boswellia serrata* containing 65% of boswellic acids (BAs) has been shown to be hepatoprotective against DOX administration in mice [151]. BAs increased Nrf-2 and HO-1 expression, and also inhibited lipid peroxidation and DNA damage. Similarly, DOX-mediated increases in markers of oxidative stress were reduced by genistein pre-treatment in mice [152]. Again, the soybean polyphenol (genistein) induced Nrf-2, HO-1, and NQO1 expression and reduced DOX-induced ROS, lipid peroxidation, and 4-hydroxynonenal (HNE) protein adduct levels. Acacetin (5,7-dihydroxy-4-methoxyflavone) was used in a recent mouse study to prevent DOX-induced cardiotoxicity [116]. The compound was given 3 days before DOX treatment and protected against cardiac dysfunction and myocardial fibrosis. Besides the induction of the Nrf-2 pathway, followed by HO-1 and SOD1/SOD2 expression, Sirt1 and pAMPK protein levels were sustained. The majority of studies described here were conducted with healthy animals and (cardio)protection of tissue as the main objective. Only a few cancer model animals were investigated, and thus the outcome on tumor size or cancer drug resistance is unknown. Another widely used CAMS is sulforaphane (SFN) which is found in cruciferous vegetables such as broccoli or Brussels sprouts. Sulforaphane is a strong Nrf-2 activator and can reduce oxidative stress in vitro and in vivo [153]. A recent review by Calcabrini et al. [153] summarized the ability of sulforaphane to sensitize tumor cells against DOX or cisplatin treatment, and to protect tissue from unintended side effects. Sulforaphane was co-administered with DOX in a tumor-bearing rat model to show a significant reduction in tumors compared to DOX treatment alone [153]. In addition, sulforaphane co-administration enhanced mitochondrial respiration, activated Nrf-2 antioxidative defense, and consequently protected rats from severe cardiotoxicity [154].

Two recent systematic reviews collected data from in vitro and in vivo studies with curcumin (CC) co-administered as a cardioprotective agent [155,156]. Curcumin is a phytochemical that is obtained from the rhizomes of *Curcuma longa* L. and it acts as an antioxidant with anti-apoptotic and anti-inflammatory effects on healthy cells. The polyphenol is a strong inducer of Nrf-2 protein and reduces NF-κB, the most important proinflammatory transcription factor. Subsequently, the DOX-induced increase in inflammatory cytokines, such as TNF-α, IL-1β, interferon gamma or COX-2, and iNOS, is reduced by curcumin co-administration [155]. In addition, a series of studies demonstrated curcumins in vitro activity against chemo-resistant breast, colorectal, leukemia, lung, and prostate cancer cell lines [157]. Only a few human trials have been conducted on curcumin as adjuvant along with gemcitabine or oxaliplatin administration (see below), but not in combination with DOX [158].

The main bioactive polyphenol from green tea is epigallocatechin-3-gallate (EGCG). Numerous studies confirmed its antioxidant and anti-inflammatory effect that is mediated by Nrf-2-activation and the inhibition of pro-inflammatory transcription factor NF-κB [159,160]. As an exception, Kweon et al. [161] found Nrf-2 inhibitory activity of EGCG at higher concentrations (300 µM) in A549 human lung adenocarcinoma cells. EGCG reduces DOX-induced cardiotoxicity in cardiomyocytes from neonatal rats when used as an adjuvant in chemotherapy [162] and enhances the anti-tumor activity of DOX in bladder cancer xenografts in mice via the reduction of the NF-κB/MDM/p53 pathway [163].

#### 3.2.3. Platin-Based Cytostatics

Platin-based cytostatics consist of a planar (cis-)coordinated platinum atom bound to four ligands, either to two chlorides and two ammine moieties (cisplatin) or chelating carboxylates (i.e., carboplatin or oxaliplatin). Cisplatin has been used as a cytostatic since the mid-1970s to fight different cancer types, including sarcomas, small-cell lung cancer (SCLC), bone cancer, ovarian cancer, muscles, and blood vessel cancer [164]. Cisplatin mostly binds to guanine residues of DNA and crosslinks single-stranded and double-stranded DNA, respectively, thereby inhibiting DNA repair and synthesis. Consequently, cell division is blocked, and fast proliferating (cancer) cells move to apoptotic cell death. It has been well documented that along with DNA modification(s), mitochondrial oxidative stress is responsible for triggering apoptosis [127,164], independent of the cisplatin-induced DNA damage [165,166]. With current cisplatin therapies, nephrotoxicity, peripheral neuropathy (PN), and ototoxicity are the main side effects that can occur, with nephrotoxicity affecting 30–40% of patients [167]. Ototoxicity is associated with the excessive generation of intracellular ROS in the cochlea [168].

Several studies show an increase in cellular ROS production during cisplatin and oxaliplatin therapy, respectively [127,169]; however, only oxaliplatin was able to induce Nrf-2 activation in Caco2 cells [170]. Another cell-based reporter gene study reported a weak induction of Nrf-2 by cisplatin [171]. Consistent with this observation, Yang et al. [14] rate anthracyclines highest in ROS production, followed by cisplatin or taxanes.

Li et al. [124] investigated several proteins and chemical compounds that (inter)act on Nrf-2 activity in ovarian cancer cells. They identified Nrf-2-activating activity by p62, SIRT5, or GPX4, which can be used to overcome cis-platin-induced drug resistance and induce cancer cell death by Nrf-2 inhibitors such as brusatol, ailanthone, or ascorbic acid.

Chemotherapy-induced peripheral neuropathy (CIPN) is widely seen with patients under cisplatin or combined cisplatin therapies. A recent study combining high-dose vitamin C (intravenous) and carboplatin–paclitaxel regimen showed an improved immune response in non-small-cell lung cancer patients [172]. Two recent meta-analyses and a systematic review reported positive results, with vitamin E reducing CIPN incidence [18,173,174].

Nrf-2 activators derived from edible plants have been proposed to reduce the side effects of cisplatin therapies [175]. We will highlight only some of the natural products mentioned in the recent literature, which were also mentioned in the surveys listed in Section 2. In a preclinical trial, rats were treated with cisplatin, and the i.p. administration of 3 × 200 mg/kg curcumin prevented tubular necrosis and renal fibrosis [176]. A significant reduction in rat renal Nrf-2 protein levels was observed during cisplatin dosing alone. A combined regimen of cisplatin and curcumin kept Nrf-2 levels at vehicle control levels, with curcumin administration exceeding the vehicle level of Nrf-2 [176]. Howells et al. [177] examined the effect of curcumin (2 g/d, oral) in combination with a FOLFOX regimen in patients with colorectal liver metastases. Curcumin was safe and tolerable but did not improve quality of life or neurotoxic symptoms.

In Wistar rats, 24 h pre- and post-cisplatin, the administration of SFN significantly reduced the markers of oxidative stress (4-HNE and H_2_O_2_) and protected the animals from nephrotoxicity [178]. Pre-treatment with SFN before cisplatin exposure activated Nrf-2 and related target genes (i.e., GCLC and NQO1) and protected them from cisplatin-induced renal cell injury [153]. The use of melatonin as a nephroprotective adjuvant in cisplatin therapy could not be confirmed in a recent study by Karvan et al. [179].

#### 3.2.4. Taxanes

Taxanes belong to the chemical class of sesquiterpenes and were first isolated from the bark of the pacific yew tree (*Taxus brevifolia*). In 1984, paclitaxel was approved by the FDA for the treatment of ovarian carcinoma and later for breast, lung, cervical, and pancreatic cancers. Taxanes inhibit the mitosis of cells by targeting the microtubules during cell division. Peripheral neuropathy is the most common side effect of paclitaxel treatment, affecting up to 60% of all treated cancer patients [180]. In addition, neutropenia, leukopenia, nausea, vomiting, mucositis, alopecia, myalgia–arthralgia, hypotension or hypertension, bradycardia, and hypersensitivity reactions can occur [181].

Paclitaxel and other taxanes are able to induce ROS, which are partly responsible for their cytotoxic activity [181]. Alexandre et al. [182] found an increase in superoxide radicals and H_2_O_2_ in paclitaxel-treated A549 cells, and the co-administration of N-acetyl-cysteine (NAC) or glutathione diminished the toxic effect of the therapy. Paclitaxel induces mitochondrial ROS in peripheral sensory nerves that is followed by the induction of SOD and GPx, suggesting the activation of Nrf-2 [183]. Tumor cells might develop ROS-dependent chemo-resistance after paclitaxel treatment. The redox-sensitive transcription factors Nrf-2 and HIF-1α are involved and neovascularization mediated by vascular endothelial growth factor (VEGF) maintains cell resistance [184]. To sensitize paclitaxel-induced chemo-resistant cells, a series of studies suggest the Nrf-2 pathway as a main target [111,181]. 

The protecting role of antioxidants to reduce paclitaxel-induced side effects is not evident since clinical trials with AO and paclitaxel monotherapy are scarce and contradictory. A recent phase II trial with 140 cancer patients found no protecting role of vitamin E in the prevention of peripheral neuropathy [185]. An earlier study with 32 patients found a lower incidence of paclitaxel-induced neurotoxicity in the vitamin E supplement arm [186]. High-dose intravenous vitamin C seems to have neuroprotective effects; however, the phase II clinical trial included patients (*n* = 38) with a carboplatin–paclitaxel combination therapy [172].

#### 3.2.5. Alkylating Anticancer Drugs

There are a series of DNA alkylating agents which belong to the class of so-called nitrogen mustards. For instance, cyclophosphamide is used as a chemotherapeutic agent in large granular lymphocyte (LGL) leukemia and as an immunosuppressant [187]. Others such as the second-generation alkylating drug temozolomide (TZM) is widely applied in glioblastoma because the molecule easily crosses the blood–brain barrier with a cerebrospinal fluid concentration of about 20% of the plasma level [188]. TZM has shown to upregulate ROS production in glioblastoma cells and in non-small-cell lung cancer cells [189]. A recent review discussed the use of TZM in cancer therapy and the role of chemically induced ROS as mediators of tumor cell apoptosis or acquired tumor chemo-resistance [190]. Animal studies with cyclophosphamide (CP) in rats show an increased expression of a series of pro-inflammatory mediators such as NF-κB, inducible nitric oxide synthase (iNOS), cyclooxygenase-2 (COX-2), TNF-α, and IL-1β [191].

Bael et al. [192] conducted a phase II trial with arsenic trioxide and ascorbic acid to treat patients (*n* = 11) with advanced melanoma. Since no response in the patients was observed, the study was closed early. In recent years, no other human study was carried out in relation to alkylating anticancer drugs.

#### 3.2.6. Radiation Therapy

Besides surgery and chemotherapy, radiotherapy is the most important approach in cancer treatment, especially for glioma, prostate, and neck cancer. Radiotherapy might be applied preoperatively in rectal cancer or post-surgery in breast cancer. A typical regimen of radiation therapy consists of fractionating a daily dose of 1.8–2 Gray (Gy) for 5 days a week. At that time, single- and double-strand breaks occur in the DNA, the number of which depends on the applied radiation dose. In addition, high-energy photons can produce highly reactive radicals and ROS that attack DNA and induce ROS-dependent apoptosis [193].

There is a strong correlation between Nrf-2 expression and radioresistance of lung, prostate, and nasopharyngeal cancer cells [105]. As such, Nrf-2 downregulation or inhibition of nuclear translocation is a main goal of adjuvant therapy with phytochemicals. Cell culture experiments with Nrf-2 inhibitors show an increase in cellular ROS and reduced cell proliferation due to enhanced apoptosis [194]. Interestingly, the Nrf-2 activator EGCG induced the nuclear import of Nrf-2 and enhanced apoptosis and autophagy in HCT-116 colon cancer cells [195].

### 3.3. Recent Clinical Trials with Combined Cancer and Adjuvant Antioxidant Therapy

We found that most of the clinical trials with antioxidants or supplements as adjuvants were applied to radiation or radio-chemotherapy regimens (Table 2). A small (*n* = 14) phase I trial with a high dose of intravenous vitamin C increased median overall survival and progression-free survival rates compared to the institutional average [196]. These promising results with high-dose ascorbate are in line with observations from clinical trials under chemotherapeutic regimens. There are contradictory results for vitamin E in relation to the improvement in radiotherapy-induced side effects in head and neck cancer patients under radiotherapy. A randomized trial with 540 patients detected an increase in all-cause mortality when vitamin E (400 IU/d) was administered during radiotherapy [197]. A recent study by Sayed et al. [198] found a significant reduction in the duration and severity of radiotherapy-induced mucositis and dysphagia when given 1000 mg/d vitamin E [198]. Similarly, clinical trials with curcumin (1.5–2 g/d) demonstrated a significant reduction in mucositis when administered adjuvant to radiotherapy [199,200]. Several phase II trials with EGCG showed significant reductions in radiation-induced dermatitis [201] and esophagitis [202], respectively. In a recent meta-analysis, the most promising results in adjuvant radiotherapy have been reported for melatonin [203]. The authors found a significant reduction in remission rates, side effects (fatigue and neurotoxicity), and 1-year survival rates.

## 4. Conclusions

The use of CAM supplements by cancer patients is widespread, with dietary supplements, vitamins and minerals, herbal remedies, and antioxidants being especially popular. In a systematic literature review, 37 studies, each including more than 1000 participants, on CAM, dietary supplement, and vitamin use among cancer patients were identified. An analysis of study results showed that the after-diagnosis use of CAM and dietary supplements, vitamins, and minerals was common among cancer patients, especially for multivitamins but also for single nutrients (e.g., vitamins A, C, and E) or specific groups of supplements (antioxidants). Dietary supplements and vitamins were taken for different reasons, but often during conventional cancer treatment involving chemotherapy or radiotherapy and in a self-decided manner without seeking medical advice by healthcare professionals. However, possible adverse effects are discussed in the literature, especially due to interactions of dietary supplements and antioxidants with cancer drugs and reduced effectiveness of conventional therapies. Possible negative consequences of dietary supplement use during cancer treatment were recently highlighted in a study by Jung et al. [48], reporting adverse outcomes of antioxidant supplementation in a large cohort of breast cancer patients. Besides the self-administered use of CAMS, we here present a brief summary of recent pre-clinical and clinical data on the use of the same chemical compounds in adjuvant chemo- and radiotherapy. Except for melatonin, the results of these studies and meta-analyses are contradictory (Table 2) [220].

More than 20 years ago, Barry Halliwell questioned the use of antioxidants in clinical trials and cohort studies since their paradoxical role as anti- or pro-oxidant led to uncertain results in former trials [221]. Each single compound used as an antioxidant supplement must be considered as an individual pharmacological agent with its own pharmacokinetics, signaling cascades, and drug–drug interactions.

For example, vitamin C acts as an antioxidant in low concentrations, but at high doses (1 g/kg body weight, intravenously) it reacts with intracellular iron and produces hydroxyl radicals [222]. In combination with chemotherapeutics, this mechanism could lead to a further increase in ROS and diminish tumor survival. Thus, a recent phase II trial with VC and carboplatin–paclitaxel on non-small-cell lung cancer found an improved tumor response rate, although the combination therapy could not overcome mutations in the Nrf-2-Keap I complex [172]. Another study with ovarian cancer patients and a VC/DOX/paclitaxel regimen showed enhanced chemosensitivity and a reduction in chemotherapy-induced side effects; however, neither the difference in progression-free survival nor the overall survival rates differed significantly between the groups [204]. A relatively low VC dose (6 g/d, oral), together with VE and beta-carotene as an adjunct to carboplatin–paclitaxel, did not reduce toxicity and tumor response rates [205]. Here, one might speculate that VE further attenuates the pro-oxidative effect of VC. In conclusion, the studies imply beneficial effects with high-dose (IV) vitamin C in adjuvant therapies or even in monotherapy, as suggested by several authors [17,223]. However, the use of VC as complementary therapy with malignant diseases is not sufficiently supported by clinical data [143].

In general, clinical trials with vitamin E as an adjuvant in chemo- or radiotherapy have been disappointing so far. Although two recent meta-analyses confirm a reduction in chemotherapy-induced peripheral neuropathy (CIPN) incidence by vitamin E [18,173], other authors see a negative influence on survival rates and a significant increase in all-cause mortality under vitamin E supplementation [174,197]. We would like to point out that vitamin E might act solely as an antioxidant, thus preventing the apoptosis-inducing ROS caused by anticancer drugs and radiotherapy, respectively.

Numerous preclinical studies on the plant compounds curcumin, EGCG, and sulforaphane as adjuvant therapies show promising results [154,157,163]. These compounds can reduce chemo-resistance in cancer and cancer stem cells, thereby increasing chemosensitivity. They reduced DOX-mediated cardiotoxicity in animal models and appeared to be safe and tolerable in human trials. Unfortunately, curcumin and EGCG have a low bioavailability, ranging between 1 and 2% of the orally administered dose [208,211], whereas sulforaphane is present in serum at 10–63% after oral intake [213]. A phase II trial with curcumin (2 g/d, oral) and a FOLFOX regimen showed no differences in quality of life (QOL) or neurotoxicity [177]. Several phase I escalated clinical trials, mostly with pancreatic cancer patients, demonstrated the safety of oral administered curcumin up to 8 g per day [158]. However, due to low patient numbers, the beneficial health effects of curcumin on survival rate or tumor progression were not clear. Two recent meta-analyses revealed a significant reduction in severe mucositis when (radio)chemotherapy is combined with a daily dose of 1.5–2 g of curcumin [199,200].

Curcumin, EGCG and sulforaphane are strong inducers of the Nrf-2-pathway with sulforaphane being the most prominent [161,200,210]. From a chemical point of view, sulforaphane is neither a typical antioxidant (phenolic hydroxyl groups are missing), nor does it show pro-oxidative structural elements. When it solely acts as a strong Nrf-2 activator in healthy cells, it may prevent them from side effects, but as discussed earlier (Section 3.2), high concentrations of SFN are able to activate Kfl9 expression and “overwrite” oxidative defense systems [119]. The question surrounding whether this biphasic property of sulforaphane leads to new therapeutic avenues needs to be answered.

Clinical trials with melatonin as an adjuvant show promising results. A reduction in chemotherapy- and radiotherapy-induced side effects, such as mucositis, fatigue, nephrotoxicity, and neuropathy, could be achieved. In addition, a recent meta-analysis observed a robust effect on cancer remission rate and 1-year survival rate [203]. The bioavailability of melatonin with 9–33% of the administered oral dose is comparatively high. Interestingly, melatonin seems to influence the Nrf-2 signaling pathway in a pleiotropic manner, where it activates the Nrf-2 pathway via melatonin receptors (MT1 and MT2) and SIRT1 and PI3K/AKT pathways in non-tumorigenic cells, and also inhibits the SGK1-mediated upregulation of Nrf-2 in tumor cells [217]. This could lead to the protection of healthy tissue during radio-chemotherapy and blocking chemo-resistance in cancer cells. Further research on melatonin in combination therapy is needed to confirm these results.

**Figure 2:**
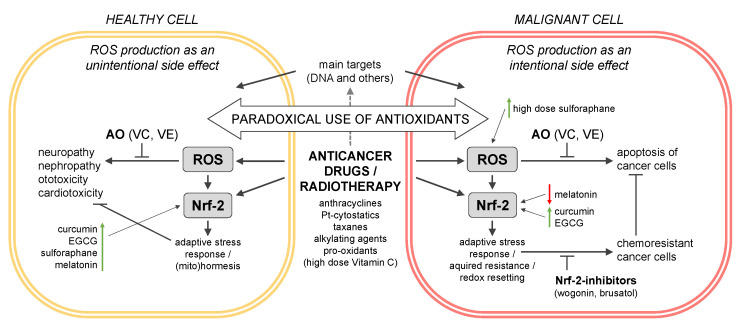
Chemo- and radiotherapy-mediated ROS induces apoptosis in malignant cells and side effects in healthy cells at the same time. The paradoxical use of antioxidants (AOs) might diminish side effects, but could also lead to chemo-resistant cancer cells (own illustration). VC: vitamin C; VE: Vitamin E; EGCG: epigallocatechin-3-gallate; ROS: reactive oxygen species; Nrf-2: nuclear erythroid-related factor-2; ↑ increase, ↓ decrease.

As we showed in this review, the Nrf-2-Keap I stress response network plays a crucial role in cancer drug resistance and the control of ROS homeostasis in healthy cells during chemotherapy (Figure 2). At the same time, chemotherapeutic drugs must hit the cancer cells with an excessive ROS production to induce apoptosis and prevent drug resistance. As to our knowledge, the impact of ROS in healthy and cancer tissue is well documented; however, the exact “dose” of ROS generation (and thus Nrf-2 activation) on the effectiveness of anticancer drugs has not been investigated or estimated so far. Moreover, the use of CAMSs as antioxidants that are alleged to diminish ROS-induced side effects could interact with the Nrf-2 signaling pathway. Again, the dosing and timing of the CAMS administration might be crucial for the success of the therapy.

As we learned from in vitro and in vivo animal studies, the co-administration of sulforaphane and curcumin seems to protect healthy tissue from the side effects of cancer drugs. The ability of these compounds to activate Nrf-2 nuclear translocation and thus stimulate the antioxidative stress response systems is a promising route to reduce side effects, such as cardiotoxicity from DOX or neuro- and nephrotoxicity from platinum derivatives [154]. As shown for a sulforaphane/DOX regimen, an additional chemo-sensitization of cancer tissue was observed. We reported several clinical trials with melatonin as adjuvant in chemo- and radiotherapy. The outcomes of side effect protection, remission rates, and cancer survival are very promising but need further confirmation.

## 5. Recommendations and Outlook

Based on our observations, we would like to conclude with several recommendations for further studies.

- Dose-dependent pharmacokinetic studies with combined CAMS along with radiation and/or chemotherapy.

Recently, Thomas et al. [224] stated that the pleiotropic effects of commonly used drugs on ROS homeostasis is an under-appreciated effect in pharmacology. Many age-related diseases such as cardiovascular and neurodegenerative diseases are associated with increased ROS production and limited defense systems against ROS. The authors describe a series of commonly used drugs such as beta-blocker, statins, oral antidiabetics, dopa and dopamine agonists, antibiotics, and anticancer drugs as modulators of ROS production. Some of these drugs boost the defense systems against oxidative stress via a mitohormetic effect and thus strengthen general health [225]. The authors conclude that dose and intervention time should be adjusted to individual oxidative levels to induce a long-lasting impact. The vitamins and phytochemicals described in this review display a wide range of pharmacological effects. Many also act on Nrf-2, the main transcription factor, to regulate ROS production. However, some of these phytochemicals show low bioavailability in humans, poor water solubility, and low intestinal absorption combined with a rapid metabolism and fast clearance, which makes it difficult to establish reasonable plasma levels. To obtain a similar plasma concentration, as described in the animal studies, IV administration (as seen for vitamin C) may be necessary. As an alternative, nano formulations of EGCG or curcumin could increase bioavailability and thus plasma levels [157].

- An establishment of stable biomarkers for drug resistance

Sun et al. [226] established a reactive oxygen scoring system based on 179 ROS-related genes in ovarian cancer patients undergoing cisplatin therapy. The authors concluded that ROS overproduction enhanced drug sensitivity and the scoring system could predict the survival prognosis of the patients [226]. Mutations in the Nrf-2-Keap I network, but also in other critical cell survival pathways, are important predictors of drug resistance and thus survival of the cancer patient.

- Large-scale studies with cancer patients taking self-administered supplements

Although there are numerous studies on the use of CAMSs among cancer patients (often with a limited number of participants), we identified a lack of large surveys and cohort studies investigating the use of dietary supplements in detail. Available studies often report on the intake of supplements during cancer treatment; however, an evaluation of possible interactions requires more specific information on the therapies, such as the anticancer drugs used. This information is also of high relevance for investigating effects of dietary supplement and antioxidant use on cancer recurrence and survival. Further, it would be interesting to compare the use of CAMSs by cancer patients in different countries, which showed huge variations in our analysis.

- Healthcare professionals need to strengthen communication with cancer patients on the use of CAMSs, especially during anticancer therapy

Based on current scientific knowledge, and considering not fully known mechanisms and interactions as well as their consequences, healthcare professionals and especially treating oncologists need to strengthen communication with cancer patients on the use of dietary supplements and antioxidants during cancer treatment. To enable information-based decision making, cancer patients need to be informed about the potential health benefits and risks of using CAM.

## Figures and Tables

**Table 2:** Recent meta-analyses and trials (both pre-clinical and clinical) with vitamins C and E, melatonin, EGCG, curcumin, and sulforaphane, including additional data on pharmacokinetics and Nrf-2 activation.

CAMS/AO	Pharmacokinetics/Bioavailability (in % of Oral Dose)	Nrf-2 Activation (++, +, −, −−) ^[a]^	CAM-Drug Combination	Clinical Trial/Meta-Analysis/Model System	Outcomes
Vitamin C (VC)	15–100 g/d (IV), Cmax: 350–400 mg/dL (20–30 mM) [204]	(+)	VC (15–100 g/d)–DOX–paclitaxel	Early-phase trial, ovarian cancer (*n* = 27)	Significant reduction in chemotherapy-induced side effects [204]
		(−)	VC (6.1 g/d) + dl-alpha-tocopherol (1050 mg/d) + beta-carotene 60 (mg/d)–paclitaxel–carboplatin	Clinical trial, non-small-cell lung cancer (*n* = 136)	No significant differences in toxicity and tumor response rate [205]
			VC (75 g/2× week)–carboplatin–paclitaxel	Phase II trial, non-small-cell lung cancer (*n* = 38)	Significant improvement in tumor response rate, improved immune response, VC administration does not overcome Keap I or Nrf-2 mutations (chemo-resistance) [172]
Vitamin E (VE)	800 mg RRR-alpha-tocopherol (oral), Cmax: 19 µg/mL (~16%) [206]	(−)	VE (200 mg gamma-tocotrienol) + pentoxifylline (2× daily)–radiotherapy	Phase II trial, pelvic cancer (*n* = 62)	No clinical benefit was demonstrated [207]
			VE (1000 mg/d) + pentoxifylline (2× daily)–radiotherapy	Randomized controlled trial, head and neck cancer (*n* = 60)	Significant reduction in duration and severity of mucositis and dysphagia [198]
			VE (400 mg/2× daily)–taxane-based chemotherapy	Phase II trial, cancer patients (*n* = 140)	No protective role in chemotherapy-induced peripheral neuropathy (CIPN) [185]
			VE (600 mg/d, oral)–Pt-based or paclitaxel	Meta-analyses (*n* = 418 and *n* = 486)	Significant lower incidence of CIPN [18,173]
			VE (400–800 mg alpha-tocopherol)–chemotherapy	Systematic review (*n* = 1941)	Reduction in oral side effects (mucositis), potential negative influence of survival rates [174]
			VE (400 IU/d)–radiotherapy	Randomized trial, head and neck cancer (*n* = 540)	All-cause mortality was significantly increased in the supplement arm [197]
Curcumin (CC)	10 g (oral), Cmax of CC: n.d., Cmax of CC conjugate: 2.3 µg/mL (~0.2%) [208] 207 mg as micelles (oral), Cmax: 412 nM [209]	(++) [210]	CC (2 g/d, oral)–oxaliplatin (FOLFOX)	Phase IIa trial, colorectal liver metastases (*n* = 18)	CC is safe and tolerable, with no differences in QOL, neurotoxicity, or CXCL1 [177]
			CC (1.5–2 g/d, oral) radio- and radio-chemotherapy	Meta-analyses, head and neck cancer (*n* = 582 and *n* = 266)	CC significantly reduced the severity of oral mucositis [199,200]
			CC/DOX	Chemo-resistant cell lines	Chemo-resistance ↓ [157]
Epigallocatechin-3-gallate (EGCG)	1200 mg (oral), Cmax: 3.4 µg/mL (~2%) [211]	(+)	EGCG (400 mg/3× daily, oral)–radiotherapy	Clinical trial, breast cancer (*n* = 10)	Significant reduction in VEGF and HGF (*p* < 0.001) [212]
	400 mg (oral), Cmax: 0.8 µg/mL (~1%) [211]	(−−) (at high (300 µM) concentration) [161]	EGCG (600 µmol/L, spray/d)–radiotherapy	Phase II trial, radiotherapy after breast cancer surgery (*n* = 165)	Significant reduction in radiation-induced dermatitis (*p* = 0.08) [201]
			EGCG (440 µmol/L/d, oral)–radiotherapy	Phase II trial, radiotherapy for stage III lung cancer (*n* = 37)	Significant reduction in radiation-induced esophagitis [202]
			EGCG/DOX	Cardiomyocytes	Cardiotoxicity ↓ [162]
			EGCG/DOX	Bladder cancer xenografts in mice	Chemosensitivity ↑ [163]
Sulforaphane (SFN)	Between 10 and 63% of oral administration [213]	(++) [156]	SFN (90 mg/d)–chemotherapy	POUDER trial	Ongoing [214]
			SFN/DOX	Rat xenografts	Chemosensitivity ↑, cardiotox.↓ [154]
			SFN/CP	Mice xenografts	Chemosensitivity ↑, nephrotoxicity↓ [178]
Melatonin (ME)	Between 9 and 33% of oral administration [215]	(++) in non-tumorigenic cells	ME (3% gel, oral mouthwashes)–radiotherapy + cisplatin–cetuximab	Phase II trial, head and neck cancer (*n* = 84)	Trend to lower incidence of severe oral mucositis [216]
		(−−) in tumorigenic cells [217]	ME (20 mg/d, 5 d)–cisplatin	Clin trial, solid tumors (*n* = 66)	Non-statistical improvement in nephrotoxicity [179]
			ME (20 mg/d, 10 d)–chemotherapy	RCT, breast cancer (*n* = 36)	Neuroprotective effect of melatonin [218]
			ME (n.d.)–radio-chemotherapy	Meta-analysis, solid tumors, (*n* = 761)	Significant reduction in remission rates, 1-year cancer survival, and side effects (fatigue and neurotoxicity), respectively [203,219]

Abbreviations: VC: vitamin C; VE: vitamin E; SFN: sulforaphane; CC: curcumin; EGCG: epigallocatechin-3-gallate; ME: melatonin; RCT: randomized clinical trial; DOX: doxorubicin; CP: cisplatin, CXCL1: chemokine (C-X-C motif) ligand 1; VEGF: vascular endothelial growth factor; HGF: hepatocyte growth factor; CIPN: chemotherapy-induced peripheral neuropathy; QOL: quality of life. ^[a]^ (++) = strong activation, (+) = activation, (−) = no effect, (−−) = inhibition. ↑ increase, ↓ decrease; n.d. no data.
